# Improving Efficiency of Clinical Studies Using a Total Digital Approach: Prospective Observational Study

**DOI:** 10.2196/18385

**Published:** 2021-02-18

**Authors:** Karin Schenck-Gustafsson, Carina Carnlöf, Mats Jensen-Urstad, Per Insulander

**Affiliations:** 1 Institute of Medicine Karolinska Institutet Stockholm Sweden; 2 Heart and Vascular Theme Karolinska University Hospital Karolinska Institutet Stockholm Sweden

**Keywords:** ECG recordings, women, palpitations, full digitalization, eAuthentication, BankID, clinical trial, mHealth, electrocardiogram

## Abstract

**Background:**

In general, most clinical studies have long recruitment periods. Signing the informed consent is particularly time-consuming when the participant must meet physically with the researchers. Therefore, introducing fully web-based techniques with the use of eAuthentication (BankID) and new digital electrocardiogram (ECG) monitoring could speed up inclusion time, increase adherence, and also reach out to more remote regions.

**Objective:**

The objectives of this study were to explore whether inclusion of a large number of participants could be realized quickly by using a total digital approach both for information and signing of informed consent, along with ECG monitoring and instant feedback on a mobile device. We also explored whether this approach can increase adherence in registration of ECG recordings and answering questionnaires, and if it would result in a more geographically uniform distribution of participants covering a wide age span.

**Methods:**

Women with palpitations were intensively studied over 2 months by means of a handheld ECG monitoring device (Coala Heart Monitor). The device connects to a smartphone or tablet, which allows the participants to obtain the results immediately. Recruitment, study information, and signing the informed consent form with the help of BankID were performed in a completely digital manner.

**Results:**

Between March and May 2018, 2424 women indicated their interest in participating in the study. On June 19, 2018, presumptive participants were invited to log in and register. After 25 days, 1082 women were included in the study; among these, 1020 women fulfilled the inclusion criteria, 913 of whom completed all phases of the study: recording ECG using the handheld device, completion of the prestudy questionnaires, and completion of the poststudy questionnaires 2 months after the ECG recordings. The dropout rate was 9%. In total, 101,804 ECG recordings were made. The mean age was 56 (SD 11) years (range 21-88 years) and 35 participants were 75 years or older. The participants were evenly distributed between living in the countryside and in cities.

**Conclusions:**

Total digital inclusion recruitment of 1082 participants was achieved in only 25 days, and resulted in a good geographical distribution, excellent adherence, and ability to reach a vast age span, including elderly women. Studies using a total digital design would be particularly appealing during a pandemic since physical contact should be avoided as much as possible.

**Trial Registration:**

ISRCTN Registry ISRCTN22495299; http://www.isrctn.com/ISRCTN22495299

## Introduction

### Background

Clinical studies often take longer to finalize than originally planned [1,2] as recruitment requires time-consuming activities such as conducting information meetings and gathering signed informed consent forms. In addition, recruitment often under-represents people living in rural areas because participants are usually recruited from metropolitan areas where research centers tend to be located. To overcome similar difficulties and limitations, some industries have successfully incorporated recent developments in software technology. For example, in Sweden, companies, banks, organizations, and authorities are communicating with and entering into agreements with individuals using BankID software via the internet. Recently, BankID has also been used in psychiatry research to collect signed informed consent [3].

A total digital approach could be used to shorten recruitment time, increase protocol adherence, and ensure representative sampling. That is, this approach could be used to more efficiently recruit participants nationally, provide study information to potential participants, collect signed informed consent forms, deliver interventions, receive and give feedback, and evaluate participants’ experiences. Specifically, the aim of this study was to investigate the use of a digital approach in the context of women using a handheld digital electrocardiogram (ECG) device to monitor palpitation symptoms (The Red Heart Study).

### Objectives

This study had four objectives: (1) to determine whether rapid inclusion of a large number of participants is possible using only a digital approach (eg, providing study information via the web and collecting signed informed consent using digital signature technology); (2) to determine whether a web-based inclusion approach would result in a geographically uniform distribution of participants; (3) to determine whether a broad age span, including the elderly, could be achieved using only a digital approach; and (4) to determine whether the participants would be more adherent to study protocols using only a digital approach.

## Methods

### Recruitment and Participants

This was a nonrandomized prospective observational study. Information about the study and a request to participate were advertised on social media platforms such as Facebook and Instagram, and mailed to the female members of the 1.6 Million Club [4], a nongovernmental organization that focuses on women’s health. In this communication, the prospective participants were informed that they would be participating in a study that collects ECG recordings using a handheld device (Coala Heart Monitor) for 60 days, and that they would be required to complete four questionnaires before the study and 60 days after the end of the study (ie, after the ECG recordings) [5]. In addition, the prospective participants were informed of the inclusion criteria: aged>18 years, experiencing at least intermittent symptomatic palpitations, ability to read and write Swedish fluently, access to a smartphone or tablet, enrolled in eAuthentication (BankID), and sufficiently fluent in digital technology to complete the questionnaires online. The exclusion criterion was previously known atrial fibrillation or atrial flutter.

### Recruitment Period

Between March and May 2018, the study was advertised and prospective participants could register their interest to take part in the study at a dedicated website. On June 19, 2018, the study website was opened for 25 days for participants to register on a first come-first served basis with the expectation that this would be enough time to recruit 1000 women according to the calculated sample size of the Red Heart Study. If this goal was not met, the recruitment period would be extended. During the same visit to the website, the prospective participants signed an informed consent form; provided demographic information; and completed four questionnaires regarding their anxiety, symptoms, depression, and health-related quality of life.

BankID, a Swedish eAuthentication electronic identification document, is comparable to a passport, driver’s license, or other physical identification document, and is considered to be secure. BankID enables companies, banks, organizations, and authorities to identify and enter into agreements with private individuals via the internet. The Swedish BankID system consists of a security program downloaded from a bank to a computer, mobile phone, or tablet. BankID is downloaded from a bank’s online platform for use with a mobile phone or tablet, or can be mailed to the individual’s home in the form of a smart card for use with a computer. At the end of 2018, up to 7.9 of the 9 million possible bank customers in Sweden were using BankID [6].

ECG monitoring with instant feedback on a cell phone or tablet was performed using the Coala Monitor described elsewhere [5]. In this study, Swedish BankID was used to confirm the identity of the participants before they read the study information, signed the informed consent form, and completed the questionnaires [3]. Using the demographic information provided by the participants, we determined the number of participants living in metropolitan areas and the number of participants living in nonmetropolitan areas. In Sweden, the largest metropolitan areas surround the three largest cities: Stockholm, Gothenburg, and Malmö. The results were compared with the actual distribution of the Swedish population (data retrieved from Statistics Sweden) [7]. 

### Statistical Analysis

Only descriptive analyses were used to summarize the sample and study variables, which are presented as mean (SD) or n (%). 

### Ethics

The investigation conformed to the principles outlined in the 2013 revised Declaration of Helsinki and was approved by the Stockholm Regional Institutional Ethics Committee

(Dnr: 4-84/2018) [8]. The study was performed in accordance with the international conference on Harmonization in Good Clinical Practice guidelines to protect the rights, integrity, confidentiality, and well-being of the trial subjects. All patients signed written consent forms with Swedish BankID. This study is registered as a clinical trial at ISRCTN (ISRCTN22495299).

## Results

### Participants and Recruitment

**Figure 1:**
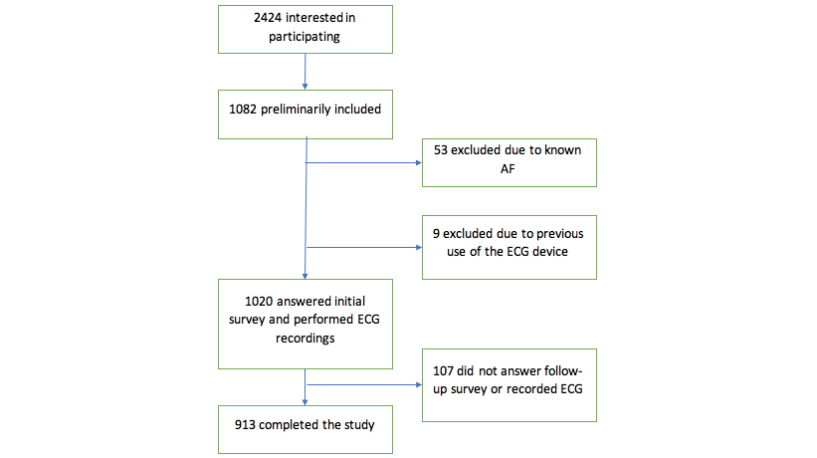
Flow chart for recruitment of the 2024 presumptive participants. AF: atrial fibrillation; ECG: electrocardiogram.

Between March and May 2018, 2424 people registered their interest in participating. On June 19, 2018, the website opened for 25 days, and presumptive participants were invited to log in and register. After 25 days (ie, on July 13, 2018), 1082 women were included in the study. After exclusion due to previous use of the ECG recording device, or previously known atrial fibrillation or atrial flutter (Figure 1), 1020 women were included. Of these, 913 completed all phases of the study: recording ECG using the handheld device, completion of the prestudy questionnaires, and completion of the poststudy questionnaires (2 months after the ECG recordings). That is, the dropout rate was 9%. In total, 101,804 ECG recordings were made. The mean age of the women was 56 (SD 11) years (range 21-88 years) and 35 participants were 75 years or older.

The participants were recruited from all over Sweden and 45% lived in metropolitan areas. Currently, 41% of the Swedish population lives in the metropolitan areas of Stockholm, Gothenburg, and Malmö (Table 1).

**Table 1:** Geographical distributions of the Swedish population and study participants.

Area	Population (N=10,230,185), n (%)	Participants (N=1089), n (%)
Stockholm metropolitan	2,371,774 (23.18)	384 (35.26)
Gothenburg metropolitan	1,039,511 (10.16)	66 (6.06)
Malmö metropolitan	739,312 (7.23)	45 (4.13)
Total metropolitan	4,150,597 (40.57)	495 (45.45)
Nonmetropolitan	6,079,588 (59)	594 (54.55)

## Discussion

### Principal Findings

For the Red Heart Study investigating underlying heart rhythms during palpitations, the use of BankID and other web-based strategies dramatically improved recruitment, information dispersal, adherence, and the collection of informed consent. In total, 1020 women were recruited in 25 days. The actual study, including daily recordings of ECGs with handheld recording devices and the completion of several questionnaires, was performed using only digital technology. Only 107 of the 1020 participants did not complete the study, resulting in a 9% dropout rate. By comparison, a recent randomized controlled trial of myocardial infarction that offered cognitive behavioral treatment for depression and anxiety had a high dropout rate and low adherence, with 46% of the participants not completing the study, mainly because they were unable or unwilling to use the internet or a mobile phone [9].

In addition, the use of digital recruitment in our study resulted in a uniform geographical distribution of participants with respect to the actual population distribution in Sweden, and an even distribution of the age of participants, who were mainly middle-aged but with a large range spanning from 21 to 88 years. Furthermore, the elderly participants managed well with eAuthentication and the ECG monitoring device connected to a smartphone or tablet.

Previously, eAuthentication using BankID has been used to collect signed informed consent as reported by Nilsson et al [3]. Very recently, BankID has been used to access eHealth platforms, mostly in psychiatric studies, as well as in lifestyle change programs for patients with diabetes and other diseases [10-12]. However, this study used eAuthentication in the context of a total digital protocol to improve adherence, representative distribution of participants, and speed of the recruitment process.

Web-based study information and informed consent may have an advantage compared to standard person-to-person meetings, as it offers the presumptive participant more time to digest, reflect, and fully understand both the study interventions and the informed consent. For example, a recent study found that patients often misunderstand the meaning of informed consent as it pertains to person-to-person appointments [13]. However, we did not compare comprehension between reading information and informed consent online and at a person-to-person meeting.

Swedish bank data reveal that 90% of BankID users are between 21 and 60 years old [6]. This percentage is likely to increase in the near future, making it possible to run fully digitalized studies, including eAuthentication, that include the elderly. In Sweden, the same proportion of women as men use Bank ID.

To date, the recruitment of study participants has been restricted to subjects living close to a research center, as the dispersal of information and informed consent often require a person-to-person interaction. As most research centers are located in metropolitan areas, subjects living in rural areas are often under-represented in trials. Using a web-based recruitment method, which included collecting signed informed consent, we were able to recruit and include subjects from all of Sweden, including less densely populated areas in the most northern parts of the country. This fully digital method also likely resulted in a more unbiased selection of subjects regarding socioeconomic status. Moreover, these digital protocols have the potential to improve stratification regarding age, sex, income, education, concomitant diseases, and drug therapies. That is, a suitable questionnaire can easily be included in a web-based model to help stratify participants. Furthermore, this digital approach to studies has the potential to save a substantial amount of money as it minimizes travel expenses, staff salary, room facilities, and compensation to participants for their time.

Some studies have found success in using digital technology to recruit participants and collect data. For example, in 2003, an internet clinical trial of 205 osteoarthritis patients randomized to either glucosamine or placebo (1200 patients applied online to take part in the study) concluded that their approach saved money, but the study was delayed because the patients needed to sign a physical written consent form and deliver their medical records to the study coordinator [14].

### Strengths and Limitations

It is reasonable to assume that the speed of recruitment in this study would not have been possible using traditional person-to-person meetings to disperse information and collect signed informed consent. Moreover, participants, including the elderly, had no problems with the digital approach. They were able to receive information, understand the study, and provide signed informed consent using BankID in this fully web-based study.

Only women with a smartphone, tablet, or computer and BankID were eligible for inclusion. Consequently, selection bias may be present as individuals uncomfortable using these digital technologies may have self-selected out. Moreover, a previous study showed that people who agree to participate in digital studies, compared to those who do not, are younger; mostly male; and have better health, quality of life, socioeconomic status, and digital skills [15]. Therefore, our study included women of all ages. Studies using a total digital design would be particularly appealing during a pandemic, as physical contact should be avoided as much as possible. Finally, the digital study design saves money both for participants and study investigators.

### Conclusion

By using online information and eAuthentication for signing informed consent, recruitment of 1020 participants was achieved in only 25 days, which is a relatively short period. Furthermore, this digital design resulted in a geographically uniform distribution of participants compared with the actual national population distribution, and therefore our study had a more unbiased selection of subjects regarding socioeconomic status. Finally, high adherence regarding questionnaires and ECG recordings was achieved using this fully digital design.

## Abbreviations

ECG: electrocardiogram
